# Prevalence of cardiovascular disease risk factors among students of a tertiary institution in Ghana

**DOI:** 10.1002/fsn3.565

**Published:** 2018-01-12

**Authors:** Eric K. Ofori, Freda D. Intiful, Matilda Asante, George A. Asare, Patrick K. Adjei, Rebecca K. Steele‐Dadzie, Anna Amoako‐Mensah, Daniel Mensah, Seth K. Angmorterh

**Affiliations:** ^1^ Department of Medical Imaging (Radiography) School of Allied Health Sciences University of Health and Allied Sciences Ho Ghana; ^2^ Department of Dietetics School of Biomedical and Allied Health Sciences College of Health Sciences University of Ghana Accra Ghana; ^3^ Department of Medical Laboratory Sciences School of Biomedical and Allied Health Sciences College of Health Sciences University of Ghana Accra Ghana; ^4^ Department of Medicine School of Medicine and Dentistry College of Health Sciences University of Ghana Accra Ghana; ^5^ Department of Nutrition and Dietetics School of Allied Health Sciences University of Health and Allied Sciences Ho Ghana

**Keywords:** blood lipid profile, blood pressure, cardiovascular diseases, obesity, prevalence, students

## Abstract

Cardiovascular diseases (CVDs) are listed as one of the main causes of mortality and morbidity by the World Health Organization. The World Heart Federation lists overweight/obesity, blood lipid profile, and blood pressure as some of the modifiable risk factors to developing CVDs. This study sought to determine the prevalence of some of these modifiable risk factors among University of Ghana students. One hundred and twenty students were sampled for the study. Lipid profile parameters such as high‐density lipoprotein (HDL), total cholesterol (TC), and total triglycerides (TG) were measured using the Vitros 5‐IFS chemistry analyzer (NY, USA). The Friedewald's equation was used to determine low‐density lipoprotein (LDL) levels. Anthropometric indices such as height and weight were measured following standard protocols. Body mass index (BMI) was calculated in kg/m^2^ using the height and weight measurements. The students were then categorized into underweight, normal, overweight, and obese according to their BMI. Blood pressure measurements were also taken. The mean age of the students was 30.04 ± 7.99 years. A total of 4.2%, 30%, and 67.5% had TG, TC, and LDL, respectively, above normal recommended ranges. Low HDL levels were observed in 32.5% of the students. About 45% had high systolic blood pressure and 32.5% with high diastolic blood pressure. In all, the risk factors studied contributed to about 95% of the variance in explaining the risk of developing CVDs. The study concludes that the cardiovascular risk factors assessed are prevalent among the students and therefore steps must be taken to address the increase in prevalence.

## 1 INTRODUCTION

The World Health Organization (WHO) lists cardiovascular disease (CVD) as one of the main causes of mortality and morbidity in the world. In 2008 alone, the WHO estimated that about 17.3 million people died as a result of CVDs. Out of this number, more than 80% of the deaths took place in developing countries (WHO, 2013). It is estimated that by the year 2020, it would account for at least 32% of mortality worldwide (Mackay, Mensah, & Mendis, 2004). In Ghana, it is ranked as the leading cause of death (de‐Graft Aikins, Addo, Ofei, Bosu, & Agyemang, 2012). In a study that focused on a 5‐year review of autopsy cases carried out at the leading hospital in Ghana (Korle‐bu Teaching Hospital) between 2006 and 2010, CVDs accounted for one fifth of all causes of death (Sanuade, Anarfi, Aikins, & Koram, 2014). The onset of CVDs is largely attributed to some risk factors which if controlled can prevent or delay the occurrence of this disease. Risk factors such as gender, hereditary, and age are regarded as unmodifiable, and therefore, nothing can be done about their contribution to the onset of CVDs. The World Heart Federation lists hypertension, tobacco use, diabetes, physical inactivity, unhealthy diet, overweight/obesity, and blood lipids as modifiable risk factors (World Heart Federation, 2013). These modifiable factors can be manipulated to delay one's chances of developing CVDs (WHO, 2005). In the United States, it is estimated that over 50% of young adults have at least one factor that places them at risk of developing a heart disease (Arts, Fernandez, & Lofgren, 2014). In Ghana, even though there is a perceived rise of CVD cases, the extent to which young people are at risk is not well documented. In the quest to reduce morbidity and mortality as a result of CVD, it is imperative to focus on addressing the risk factors in the young. This is because the onset of CVDs is not a sudden occurrence but happens as a result of long‐time exposure to the various risk factors. Many are exposed to the risk factors right from childhood through adolescence to adulthood (Reilly & Kelly, 2011). Therefore, focusing on tertiary students is one of the ways by which these factors can be assessed among the young. This study therefore aimed to determine the prevalence of some modifiable cardiovascular risk factors among students in a tertiary institution.

## 2 METHOD

### 2.1 Subjects/study design

The study employed the cross‐sectional descriptive approach. One hundred and twenty final year undergraduate students of the University of Ghana were recruited into the study. Students were recruited into the study after researchers received a verbal consent from them. This was after the protocol of the study had been explained to them. Eligible students were selected through cluster and systematic sampling techniques. Ethical clearance was obtained from the Ethics and Protocol Review committee of the School of Biomedical and Allied Health Sciences of the University of Ghana.

A structured questionnaire was used to obtain information about their demographics. Information obtained included their ages, marital status, and their programs of study.

### 2.2 Anthropometric measurements

The weights of subjects were taken in minimum clothing with the HBF‐516 Body Composition Monitor and Scale (IL, USA). The same instrument was used in estimating visceral fat. Standard protocols were followed in taking their heights to the nearest 0.1 cm using the Seca Stadiometer (Hamburg, Germany). Height and weight measurements were used in computing the body mass index (BMI) of the students. The following categorization was used in classifying them: underweight (<18.5 kg/m^2^), normal (18.5–24.9 kg/m^2^), overweight (25.0–29.9 kg/m^2^), and obese (≥30.0 kg/m^2^) (WHO, 1995).

### 2.3 Determination of blood pressure and lipid profile

Subjects were made to sit in a relaxed and comfortable position before taking blood pressure measurements. The Omron blood pressure monitor (IL, USA) was used to take blood pressure measurements. All measurements were taken on the right arm. Blood pressure levels (in mmHg) were categorized as follows: *systolic* (≤120, acceptable; >120, unacceptable) and *diastolic* (≤80, acceptable; >80, unacceptable) (Bimenya et al., 2005).

Lipid profile was assessed by drawing 3 ml of venipuncture blood from subjects into gel separator tubes. Blood samples were centrifuged at 3,000 rpm for 10 min and serum samples used for the analysis. Lipid profile parameters assessed were total cholesterol (TC), high‐density lipoprotein cholesterol (HDL‐C), and triglycerides (TG). The Vitros 5‐IFS chemistry analyzer (NY, USA) was used in analyzing the lipid profile of the subjects. The amount of low‐density lipoprotein cholesterol (LDL‐C) in each serum was obtained using the Friedewald's equation: [LDL‐C] = [TC] − [HDL‐C] − ([TG]/2.2) (Freidewalds, Levy, & Fredrickson, 1972). The ranges (in mmol/L) were categorized as follows: *total TG* (≤1.7, acceptable; >1.7, unacceptable); *TC* (≤5.2, acceptable; >5.2, unacceptable); *LDL* (≤2.6, acceptable; >2.6, unacceptable); and *HDL* (≤1 [men], unacceptable; >1 [men], acceptable; ≤1.3 [women], unacceptable; >1.3 [women], acceptable).

The ratio of TC to HDL was calculated as the cardiovascular risk ratio (Millán et al., 2009).

### 2.4 Data analysis

Data obtained were analyzed using SPSS version 17. Data were summarized using means and standard deviations. Differences in means of continuous variables between males and females were determined by *t* tests. Pearson's correlations were used to ascertain relationship between the cardiovascular risk factors. The extent to which the variables contribute to the development of cardiovascular risk was assessed using multiple regressions.

## 3 RESULTS

The students in the study were all final year undergraduate nursing students offering general nursing, midwifery, pediatric nursing, community nursing, and mental nursing. The mean age was 30.04 ± 7.99. About 58% of them were single and the rest married (39%) or divorced (2.5%). Table 1 shows the background characteristics of the students.

**Table 1:** Background characteristics of students

Variable	Male (*n* = 20)	Female (*n* = 100)	Total (*n* = 120)	*p*‐value
Age (years), mean ± *SD*	26.65 ± 5.69	30.72 ± 8.23	30.04 ± 7.99	.037^a^
Marital status, *N* (%)				
Single	17 (85.0)	53 (53.0)	70 (58.3)	
Married	3 (15.0)	44 (44.0)	47 (39.2)	
Divorced	0 (0.0)	3 (3.0)	3 (2.5)	
Program of study, *N* (%)				
General nursing	5 (4.2)	11 (11.0)	16 (13.3)	
Midwifery	0 (0.0)	34 (34.0)	34 (28.3)	
Pediatric	2 (1.7)	28 (28.0)	30 (25.0)	
Community health	4 (3.3)	27 (27.0)	31 (25.8)	
Mental health	9 (7.5)	0 (0.0)	9 (7.5)	
BMI (kg/m^2^), mean ± *SD*	22.08 ± 3.02	27.22 ± 5.74	26.37 ± 5.71	.001^a^
Lipid profile (mmol/L), mean ± *SD*				
TG	0.90 ± 0.39	0.81 ± 0.33	0.82 ± 0.34	.260
TC	4.58 ± 1.08	4.91 ± 1.01	4.86 ± 1.02	.190
HDL	1.20 ± 0.22	1.46 ± 0.25	1.42 ± 0.27	.001^a^
LDL	2.97 ± 1.10	3.08 ± 0.94	3.06 ± 0.96	.637
Visceral fat, mean ± *SD*	4.70 ± 3.01	5.93 ± 2.33	5.72 ± 2.48	.040^a^
Blood pressure (mmHg)				
Systolic	132.15 ± 15.76	118.03 ± 11.29	120.38 ± 13.18	.001^a^
Diastolic	78.20 ± 7.49	76.41 ± 8.84	76.71 ± 8.23	.390
Cardiovascular risk	3.95 ± 1.24	3.44 ± 0.87	3.52 ± 0.96	.027^a^

a *t* test significant at *p* < .05.

The summary of the prevalence of some modifiable risk factors of CVDs is presented in Table 2. More than 50% of the students were either obese or overweight (53.4%). About 96% of the students had TG levels in the normal range. Thirty percent showed TC levels higher than normal which places them at a greater risk of developing CVDs. Students who had serum levels of LDL above the normal recommendation were high (67.5%). Serum recommended levels of HDL were observed in 67.5% of the students. Students who recorded abnormal systolic and diastolic pressures were 45% and 32.5%, respectively. When the risk for developing CVD was assessed, 12.5% of the students had increased risk of developing the disease.

**Table 2:** Prevalence of cardiovascular risk factors among students

Variable	Male(*n* = 20)	Female(*n* = 100)	Total(*n* = 120)	*p*‐value
BMI (kg/m^2^),^b^ *n* (%)				
Underweight	1 (0.8)	0 (0.0)	1 (0.8)	—
Normal	15 (12.5)	40 (33.3)	55 (45.8)	
Overweight	4 (3.3)	34 (28.3)	38 (31.7)	
Obese	0 (0.0)	26 (21.7)	26 (21.7)	
TG (mmol/L)^b^				
≤1.7	19 (15.8)	96 (80.0)	115 (95.8)	—
>1.7	1 (0.8)	4 (3.3)	5 (4.2)	
TC (mmol/L)				
≤5.2	15 (12.5)	69 (57.5)	84 (70.0)	.593
>5.2	5 (4.2)	31 (25.8)	36 (30.0)	
LDL (mmol/L)				
≤2.6	9 (7.5)	30 (25.0)	39 (32.5)	.191
>2.6	11 (9.2)	70 (58.3)	81 (67.5)	
HDL (mmol/L)				
≤1 (men)	12 (10.0)	27 (22.5)	39 (32.5)^c^	.004^a^
≤1.3 (women)				
>1 (men)	8 (6.7)	73 (60.8)	81 (67.5)^d^	
>1.3 (women)				
Blood pressure (mmHg)				
*Systolic*				
≤120	13 (10.8)	60 (50.0)	66 (55.0)	
>120	7 (5.8)	40 (33.3)	54 (45.0)	
*Diastolic*				
≤80	13 (10.8)	68 (56.7)	81 (67.5)	.794
>80	7 (5.8)	32 (26.7)	39 (32.5)	
Visceral fat (%)^b^				
≤9	17 (14.2)	90 (75.0)	107 (89.2)	—
>9	3 (2.4)	10 (8.3)	13 (10.8)	
Cardiovascular risk ratio^b^				
≤4.5 (men)	15 (12.5)	90 (75.0)	105 (87.5)	—
>4.5 (men)	5 (4.2)	10 (8.3)	15 (12.5)	
≤4.0 (women)				
>4.0 (women)				

a Chi‐square test significant at *p* < .05.

b Chi‐square tests were not performed because >20% of the cells had expected counts <5.

c Total number of males and females with unacceptable HDL.

d Total number of males and females with acceptable HDL.

Table 3 shows how the students compare when they were classified according to their BMI and cardiovascular risk factors. Fifty percent of the males who had normal BMI had LDL above the recommended values, while about 47% of the women who were overweight had LDL above recommended values.

**Table 3:** Cardiovascular risk factors of students according to BMI classification

Variable	Male (*N* = 20), *n* (%)				Female (*N* = 100), *n* (%)			
	Underweight	Normal	Overweight	Obese	Underweight	Normal	Overweight	Obese
TG (mmol/L)								
≤1.7	1 (5.0)	15 (14.2)	3 (15.0)	—	—	40 (40.0)	34 (34.0)	22 (22.0)
>1.7	0 (0.0)	0 (0.0)	1 (5.0)	—	—	0 (0.0)	0 (0.0)	4 (4.0)
TC (mmol/L)								
≤5.2	1 (5.0)	11 (55.0)	3 (15.0)	—	—	34 (34.0)	17 (17.0)	18 (18.0)
>5.2	0 (0.0)	4 (20.0)	1 (5.0)	—	—	6 (6.0)	17 (17.0)	8 (8.0)
LDL (mmol/L)								
≤2.6	1 (5.0)	5 (25.0)	3 (15.0)	—	—	17 (17.0)	7 (7.0)	6 (6.0)
>2.6	0 (0.0)	10 (50.0)	1 (5.0)	—	—	23 (23.0)	27 (27.0)	20 (20. 0)
HDL (mmol/L)								
≤1 (men)	0 (0.0)	11 (55.0)	1 (5.0)	—	—	10 (10.0)	8 (8.0)	9 (9.0)
>1 (men)	1 (5.0)	4 (20.0)	3 (15.0)	—	—	30 (30.0)	26 (26.0)	17 (17.0)
≤1.3 (women)								
>1.3 (women)								
Systolic (mmHg)								
≤120	1 (5.0)	4 (20.0)	1 (5.0)	—	—	31 (31.0)	21 (21.0)	8 (8.0)
>120	0 (0.0)	11 (55.0)	3 (15.0)	—	—	9 (9.0)	13 (13.0)	18 (18.0)
Diastolic (mmHg)								
≤80	1 (5.0)	8 (40.0)	4 (20.0)	—	—	35 (35.0)	21 (21.0)	12 (12.0)
>80	0 (0.0)	7 (35.0)	0 (0.0)	—	—	5 (5.0)	13 (13.0)	14 (14.0)
Visceral fat								
≤9	1 (5.0)	15 (75.0)	1 (5.0)	—	—	40 (40.0)	34 (34.0)	16 (16.0)
>9	0 (0.0)	0 (0.0)	3 (15.0)	—	—	0 (0.0)	0 (0.0)	10 (10.0)

Correlations were computed among eight of the cardiovascular risk factors. The results are presented in Table 4. Strong correlations were observed between visceral fat and BMI (0.876, p<0.01); LDL and TC (0.967, p<0.01) and cardiovascular risk and LDL (0.773, p<0.01). Relationships between BMI and TG (0.420, p<0.01), HDL and TG (0.314, p<0.01), TC and cardivascular risk (0.651, p<0.01), diastolic and systolic pressure (0.569, p<0.01) showed moderate correlations.

**Table 4:** Relationship between the cardiovascular risk factors

	1	2	3	4	5	6	7	8	9
1. Body mass index of students	—								
2. Visceral fat of students	0.876^a^	—							
3. Systolic blood pressure	0.159	0.206^b^	—						
4. Diastolic blood pressure	0.182^b^	0.283^a^	0.569^a^	—					
5. Level of triglycerides (mmol/L)	0.420^a^	0.354^a^	0.168	0.085	—				
6. Level of cholesterol (mmol/L)	0.215^b^	0.163	0.078	0.139	0.259^a^	—			
7. HDL cholesterol (mmol/L)	0.107	0.035	−0.180^b^	0.004	−0.314^a^	0.209^b^	—		
8. LDL cholesterol (mmol/L)	0.131	0.106	0.105	0.133	0.201^b^	0.967^a^	−0.002	—	
9. Cardiovascular risk	0.091	0.109	0.187^b^	0.147	0.485^a^	0.651^a^	0.568^a^	0.773^a^	—

a Correlation is significant at the 0.01 level (two‐tailed).

b Correlation is significant at the 0.05 level (two‐tailed).

Results presented in Table 5 show that visceral fat, systolic BP, diastolic BP, TG, HDL, LDL, and BMI collectively explained 95% of the variance in cardiovascular risk. TG, HDL, and LDL explained the bulk of the predictor of cardiovascular risk since their contributions were highly significant. The contributions of visceral fat, systolic BP, and BMI were not statistically significant.

**Table 5:** Summary of regression analysis for predicting cardiovascular risk among the students

Variable	B	Beta	*R*	Adjusted *R* ^2^	*t*	significance
Constant	3.541				12.390	0.001
Visceral fat	0.007	0.019			0.412	0.681
Systolic BP	0.003	0.046			−1.682	0.095
Diastolic BP	0.007	0.060			2.172	0.032^a^
TG	0.547	0.196			7.383	0.001^a^
HDL	−1.834	−0.514			−20.808	0.001^a^
LDL	0.729	0.730			32.618	0.001^a^
BMI	−0.009	−0.055			−1.120	0.265
			.974	.945		

a Correlation is significant at 0.05 level (two‐tailed)

## 4 DISCUSSIONS

The study has provided insights into cardiovascular risk factors among the university students studied. The prevalence of obesity was high in this population (26%) in comparison with other earlier studies carried out in the country to ascertain the prevalence of obesity (Amoah, 2003; Biritwum, Gyapong, & Mensah, 2005).

The prevalence was also higher when compared to other studies carried out among university students. For instance, among students of the University of Basrah, 7.3% were found to be obese Al‐Asadi, Habib, & Al‐Naama, 2006). Also, among medical students in Greece, the prevalence was 4.3% Bertsias, Mammas, Linardakis, & Kafatos, 2003). The trend of high prevalence of overweight and obesity among the Ghanaian students is not surprising. This is because majority of the students were females and were between the ages of 25–45 years where they are usually prone to overweight and obesity (Czernichow, Kengne, Stamatakis, Hamer, & Batty, 2011). However, efforts should be made to reduce this trend because of the evidence linking obesity to increased risk of CVDs. In a meta‐analysis carried on nine cohort studies, when sex and age were adjusted for, BMI was found to be linked to increased mortality (Czernichow et al., 2011). Assessing lipid profile is one of the tools that can aid in ascertaining cardiovascular risk.

Elevated levels of TG, TC, and LDL are unacceptable in an effort to reduce CVD risk. However, higher levels of HDL are encouraged as it promotes better health (Nordestgaard & Varbo, 2014; Rader & Hovingh, 2014; Ridker, 2014). Undesirable levels of certain categories of lipids were observed among the students. More than half (67.5%) of the students had LDL levels above recommended desirable ranges. It is important to address such occurrences in the population if CVD is to be controlled. This is due to the fact that elevated LDL has been reported to increase the risk of CVD in several studies (Cantin et al., 1998; Luc et al., 2002). Blood pressure is an indicator used to measure the incidence of hypertension. High blood pressure is associated with hypertension and indicative of other CVDs (Weber et al., 2014). Prevalence of high systolic pressure (45%) and high diastolic pressure (32.5%) were quiet high among the students. Mean values obtained were comparable to studies carried out on Turkish university students (Kutlu & Memetoglu, 2013). It is unacceptable to have high systolic and diastolic pressure, therefore programs should be rolled to address such occurrence since continuous prevalence of high pressure can be a major cause for concern.

Abdominal obesity is a key indicator for developing chronic conditions such as CVDs. Accumulation of excess visceral fat is associated with elevated levels of TG, TC, and LDL with reduced levels of HDL (Despres, 2007). Other studies also indicate that it is an independent predictor of all‐cause mortality in men (World Heart Federation, 2013). The results from this study showed that 10.8% of the students had percentage visceral fat above the normal recommended ranges. This prevalence is lower than the 21.8% found among college students in Saudi Arabia (Al‐Rethaiaa, Fahmy, & Al‐Shwaiyat, 2010).

Reducing the percentage of visceral fat is imperative as it would reduce the risk of CVDs.

It is essential to note that the various risk factors interact with each other. Strong to moderate correlations was found between visceral fat and BMI, diastolic and systolic pressure, and TC and LDL levels. Managing the occurrence of one factor would have positive effects on the other. For instance, decreasing one's BMI would decrease visceral fat (WHO, 2005). A systematic approach is needed to control the risk factors studied. This is in light of the contribution of the factors studied in predicting cardiovascular risk. As much as 95% of the variance in predicting cardiovascular risk is explained by the factors studied leaving only 5% to other unexplained factors.

## 5 CONCLUSION

In conclusion, there was an increased prevalence of the cardiovascular risk factors assessed. BMI, LDL, TG, TC, HDL, and visceral fats are all important predictors to CVDs among the students studied. It is therefore vital that systematic and comprehensive programs are outlined to address its prevalence in the group studied.

## CONFLICT OF INTEREST

None declared.
